# Quantifying Intraoperative Workloads Across the Surgical Team Roles: Room for Better Balance?

**DOI:** 10.1007/s00268-016-3449-6

**Published:** 2016-03-07

**Authors:** Denny Yu, Bethany Lowndes, Cornelius Thiels, Juliane Bingener, Amro Abdelrahman, Rebecca Lyons, Susan Hallbeck

**Affiliations:** Department of Health Sciences Research, Mayo Clinic, 200 First Street SW, Rochester, MN 55905 USA; Mayo Clinic Robert D. and Patricia E. Kern Center for the Science of Health Care Delivery, Mayo Clinic, 200 First Street SW, Rochester, MN 55905 USA; School of Industrial Engineering, Purdue University, 315 N. Grant Street, West Lafayette, IN 47907 USA; Department of Surgery, Mayo Clinic, 200 First Street SW, Rochester, MN 55905 USA

## Abstract

**Background:**

Surgical performance, provider health, and patient safety can be compromised when workload demands exceed individual capability on the surgical team. The purpose of this study is to quantify and compare intraoperative workload among surgical team members.

**Methods:**

Observations were conducted for an entire surgical day for 33 participating surgeons and their surgical team at one medical institution. Workload (mental, physical, case complexity, distractions, and case difficulty) was measured for each surgical team member using questions from validated questionnaires. Statistical analyses were performed with a mixed effects model.

**Results:**

A total of 192 surgical team members participated in 78 operative cases, and 344 questionnaires were collected. Procedures with high surgeon mental and physical workload included endovascular and gastric surgeries, respectively. Ratings did not differ significantly among surgeons and residents, but scrub nurses physical demand ratings were 14–22 (out of 100) points lower than the surgeons, residents, and surgical assistants. Residents reported the highest mental workload, averaging 19–24 points higher than surgical assistants, scrub nurses, and circulating nurses. Mental and physical demands exceeded 50 points 28–45 % of the time for surgeons and residents. Workload did not differ between minimally invasive and open techniques.

**Conclusion:**

The workload questionnaires are an effective tool for quantifying intraoperative workload across the surgical team to ensure mental and physical demands do not exceed thresholds where performance may decrease and injury risk increase. This tool has the potential to measure the safety of current procedures and drive design of workload interventions.

## Introduction

The operating room (OR) is a complex environment that can create a high physical and cognitive workload for the surgical team [1–3]. Several studies have suggested that gaps between required workload (e.g., patient complexity, minimally invasive technique, and time pressure) and available capacity (e.g., inexperienced team members) not only impair performance but may also play a role in the occurrence of errors and adverse patient outcomes [2, 4–9]. Surgical team members under high *cognitive workloads* are less effective at adapting to unexpected work demands (e.g., intraoperative instrument malfunctions or complications) [10–13]. High *physical demands* (e.g., difficulty exposure or positioning) can affect motor control and have been associated with inadvertent tissue injuries [14]. They also contribute to musculoskeletal injuries reported by 70–100 % of minimally invasive surgeons as well as about 50 % of surgical technicians and assistants who spend prolonged periods in positions that are physically taxing [15–26]. Cumulative exposures to high cognitive and physical workloads may also impact career longevity [27–33].

There is a need to quantify current intraoperative workloads to identify areas where workload exceeds thresholds that may impact performance or musculoskeletal health. Literature suggests workload thresholds of 40 ± 10 (out of 100) in aviation combat tasks [34]. In healthcare, two separate studies by Mazur et al. suggested threshold of 50–55 (out of 100) is the point at which performance in clinical tasks decline and clinical errors become more common [35, 36]. Additionally, several studies in laparoscopy showed a positive relationship between mental workload and performance errors, e.g., tissue injuries and instrument positioning [14, 37]. Finally, observer-based, biomechanics, and psychophysical studies have demonstrated links between physical demand and injury risk. In addition, a recent study using the NASA-TLX questionnaire found that higher injury risks were associated with residents reporting physical demands >50 during laparoscopic skills tasks [38]. Although more rigorous studies are needed to establish workload thresholds, these preliminary workload guidelines have the potential to optimize cognitive and physical workload and its distribution across the surgical team.

As operations become more complex and require more technology, the mental and physical demand on surgeons and their teams will likely increase. To understand if surgical team members are able to excel under additional workload and whether there is room for better workload balance, we need to measure current workload and validate a method for monitoring workload as different systems are implemented or altered. The purpose of this study is to quantify and compare workload among surgical team members across different surgical techniques and specialties.

## Materials and methods

Data were collected at a large non-profit teaching hospital for this Institutional Review Board approved study (ID13-004027) between September 2013 and February 2014. Thirty-three surgical teams participated and workload data were collected on all procedures that occurred during one operating day for each participating surgeon and their team (participating roles in Table 1). Procedure type, surgical technique (i.e., open, minimally invasive surgery (MIS), or robotic), surgical specialty (i.e., colorectal, general, gynecology, vascular, and with specialties with <2 surgeons categorized as “other”), and surgical duration, defined as incision to close, were collected for each surgical case.

**Table 1:** Definition of role abbreviations and descriptions of surgical team member roles

Abbr. role	Description of roles and example observations from this study
Anes^a^	Anesthesiologist and Anesthesia Resident: supervises and administers anesthesia
CRNA	Certified registered nurse anesthetist: administers anesthesia under the supervision of an attending anesthesiologist
CN^b^	Circulating nurse: the CN handles anything not sterile including ensuring patient safety and comfort during induction and after the procedure, opening instruments for the sterile field, answers the phone, and fills out paper work
CST^b^	Certified surgical technician (scrub nurse): Ensures surgical instruments are available, counted, and handed to the surgeons
CSA^b^	Certified surgical assistant (Surgical First Assistant): Assists the surgeon during the procedure including operating laparoscope, robotic assisting, closing incisions
Resi	Resident and Fellows: typically a surgical trainee with one to six years of post-graduate experience. Duties during surgery ranges from observation to assisting surgeon during the procedure
Surg	Surgeon: Performs and supervises the procedure

^a^One out of the four anesthesiologist participants was an anesthesia resident

^b^Roles are further described by the Association of periOperative Registered Nurses (AORN)

### Assessment of workload

**Fig. 1:**
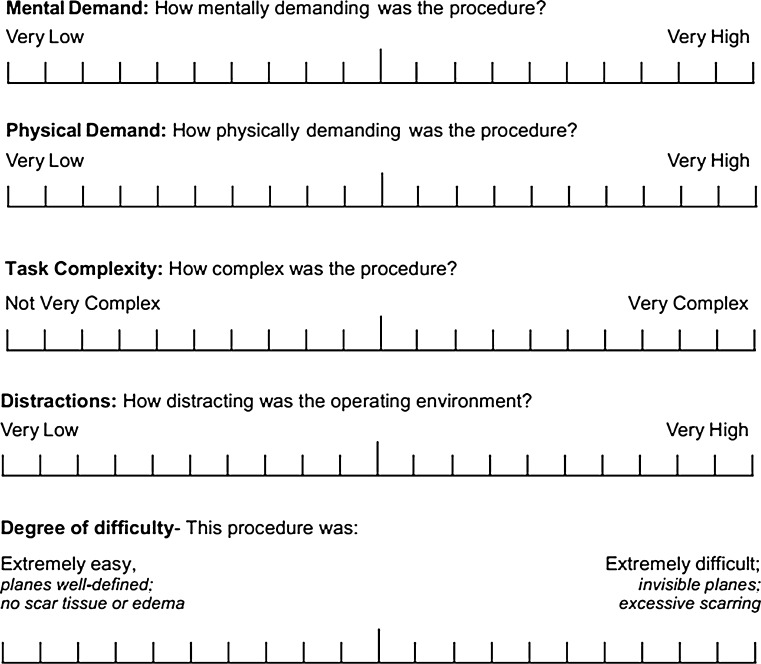
Questionnaire with five subscales (first four questions from SURG-TLX and last question from GOALS) was used to quantify workload among surgical team members

Individual workload was quantified using questions (Fig. 1) from previously validated survey questions, i.e., Surgical Task Load Index (SURG-TLX) and Global Operative Assessment of Laparoscopic Skills (GOALS) [39–41]. A modified version of the validated SURG-TLX questionnaire was used with the addition of question from GOALS to increase its relevance to measuring intraoperative workload. Adaption of SURG-TLX is a well-used technique, and this modified questionnaire is previously published and still allows for comparison of subscales across other studies [42, 43]. The resulting questionnaire (Fig. 1) was administered to each surgical team member (Table 1) immediately after every surgical procedure. Participant’s rating for each subscale (Fig. 1) is reported as out of 100 points.

### Data analysis

Data were de-identified and aggregated in a Research Electronic Data Capture (REDCap) database and analysis was performed with SPSS Statistics 22.0 for Windows (IBM™). Comparison of surgical duration between surgical techniques was performed with the Mann–Whitney *U* Test. For each dependent variable on the questionnaire, a mixed effects model was used with full-factorial combinations of case factors (i.e., surgical team role, surgical technique, and specialty). Procedure time was included as a covariate to adjust for the length of procedure. To account for covariance with respect to participants working on the same surgical case, surgical case was modeled as a random effect. Post hoc tests for multiple comparisons were performed with Bonferroni correction. All statistical tests were performed with *α* = 0.05.

## Results

A total of 192 surgical team members from the 33 surgical days were gathered. Team roles are defined and described in Table 1. Three hundred and forty-four questionnaires were collected from 78 unique surgical cases across different surgical techniques and specialties (Table 2), and sample sizes for each dependent variable are shown in Table 3. The average number of participants completing the questionnaire in each surgical case was 4 ± 2 individuals. Operative days consisted of up to four cases per surgical team. Operative times were 118 ± 67 min for MIS and 176 ± 173 min for open cases and did not differ significantly (*p* = 0.21) for our sample of 78 cases (Table 2). Surgical duration had a positive association (*p* < 0.05) with each workload subscale; parameter estimates were lowest for distractions (0.03 points/minute) and highest for degree of difficulty (0.09 points/minute). No significant interactions were observed among the predictors (*p* > 0.05), e.g., effect of the role on the rated workload does not depend on the surgical technique and specialty. Thus, results will report the main effects models, adjusted for surgical duration and random effects of individual case, for each dependent variable.

**Table 2:** Description of the operative duration and unique participants categorized by surgical technique and specialty

		Time (minutes)	# of Participants^a^							
	# Cases	Mean ± SD	Anes	CRNA	CN	CST	CSA	Resi	Surg	Total
All	78	160 ± 151	4	12	38	35	26	45	32	192
Technique										
Open	55	176 ± 173	2	10	31	26	19	37	28	
MIS	21	118 ± 67	3	3	13	13	11	19	13	
Robotic	2	147 ± 40	1	0	1	1	1	1	1	
Specialty										
Colorectal	13	152 ± 109	0	2	5	5	3	9	4	
General	39	148 ± 132	4	5	19	18	17	21	17	
Gynecology	9	128 ± 74	1	3	5	3	3	5	4	
Other^b^	11	233 ± 263	0	2	6	6	1	9	4	
Vascular	6	171 ± 171	0	0	6	3	4	5	3	

^a^Number of participants in “All” refer the number of unique participants. Sum of participants in Technique (and Specialty) may be greater than number in “All” row if participant performed in more than one technique during the study. E.g., if Surgeon #1 performed both Open and Laparoscopic during this study

^b^Surgical specialties with two or less participating surgeon were categorized as “Other” and included Otorhinolaryngology, Pediatric, Thoracic, and Urology

**Table 3:** Number of responses by role

	Anes	CRNA	CSA	CST	Resi	RN	Surg	Total
Mental demand	9	17	42	61	78	61	62	330
Physical demand	9	17	41	59	78	59	61	324
Complexity	9	17	42	58	78	61	61	326
Distractions	9	17	41	59	78	60	62	326
Difficulty	4	11	41	60	78	62	61	317

**Fig. 2:**
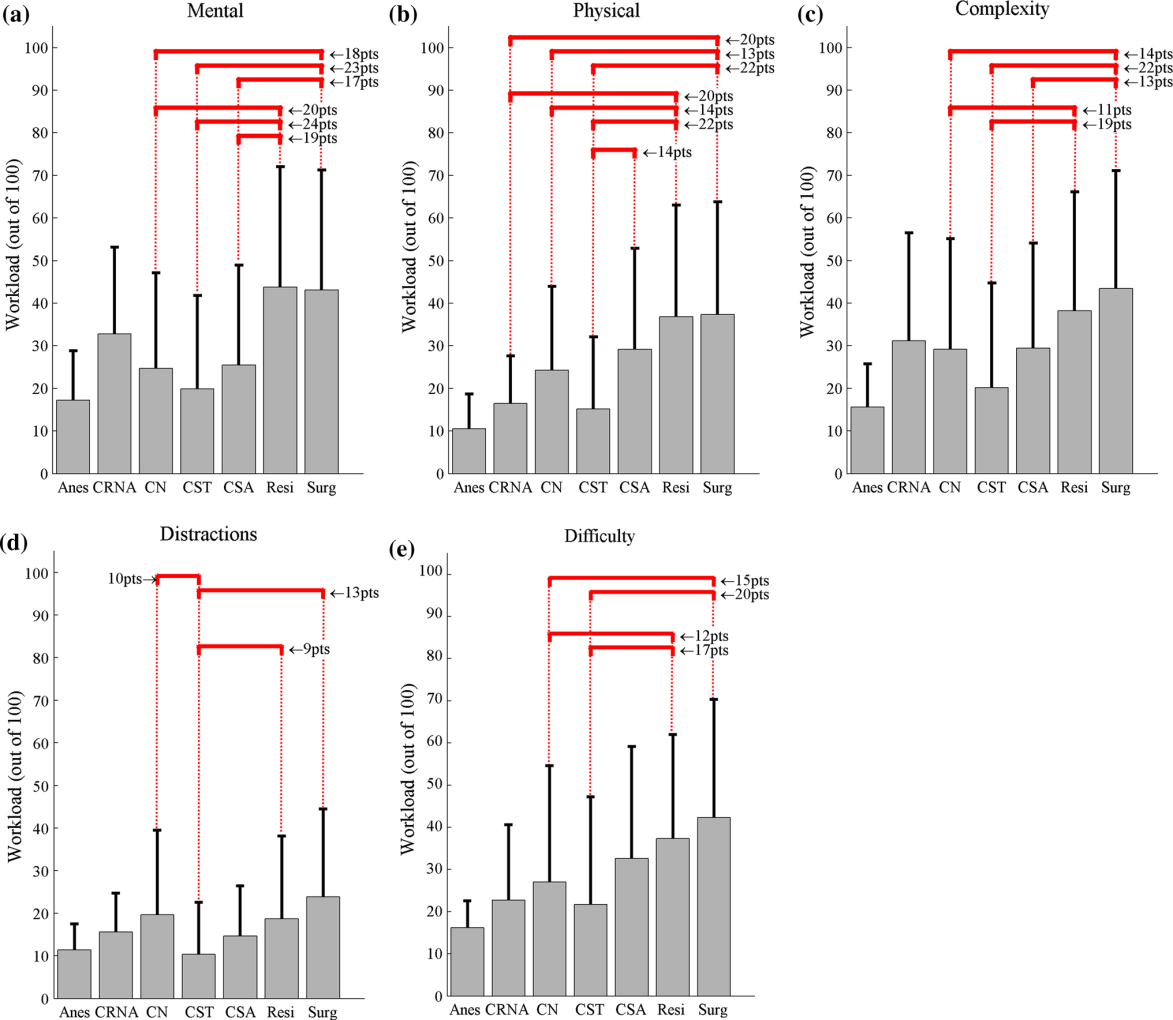
Mean and standard deviation for subscales from SURG-TLX (**a**–**d**) and degree of difficulty question from GOALS (**e**) for all questionnaires stratified by role with brackets indicating significant differences between roles (*p* < 0.05) and adjacent text showing difference in estimated marginal means

Mean ratings (out of 100) for mental demand ranged from 17 ± 12 to 44 ± 28 and ratings were highest for residents and lowest for anesthesiologists (Fig. 2a). Mean ratings for physical demand ranged from 11 ± 8 to 37 ± 26, and ratings were highest for surgeons and lowest for anesthesiologist. Mean ratings for distractions were the lowest among all subscales and ranged from 10 ± 12 for the CSTs to 24 ± 21 for the surgeons. In addition to the SURG-TLX workload measurements, mean self-rating for the degree of surgical difficulty ranged from 16 ± 6 (anesthesiologists) to 42 ± 28 (surgeons).

Surgical team role had a significant effect on all workload subscales (Fig. 2). On average, mental demand, physical demand, complexity, distraction, and difficulty were rated 9–24 points higher (*p* < 0.05) for surgeons and residents than CSTs and CNs. CSAs rated mental demand 17–19 points less (*p* < 0.005) than surgeons and residents (Fig. 2a) and experienced 14 points higher physical demand (*p* < 0.05) than CSTs (Fig. 2b). Physical demands were rated 20 points lower for CRNAs than surgeons and residents (*p* < 0.01). Distractions were rated 9–13 points lower (*p* < 0.05) by CSTs than CNs, residents, and surgeons (Fig. 2d). Specialty did not have a statistically significant impact on workload. However, mental workload in general surgery trended 14 points lower than gynecology (*p* = 0.07), and physical workload in general surgery trended 7–11 points lower than colorectal, gynecology, and vascular specialties (*p* = 0.19–0.32).

An example procedure with high mental workload for surgeons included endovascular angioplasty cases with angiogram (*n* = 3 cases, mental demand = 63 ± 6 points and physical demand = 40 ± 30 points). High physical workloads were observed for gastric surgery, i.e., gastrectomy and gastroplasty, (*n* = 4, mental = 45 ± 28 and physical = 58 ± 28), subtotal colectomy (*n* = 4, mental = 44 ± 30 and physical = 59 ± 30), and enterocutaneous fistula takedown (*n* = 2, mental = 85 ± 11 and physical = 85 ± 11). Examples of lower workload cases included cholecystectomy (*n* = 4, mental = 10 ± 29 and physical = 10 ± 27) and inguinal hernia repair procedures (*n* = 5, mental = 28 ± 34 and physical = 31 ± 27).

**Fig. 3:**
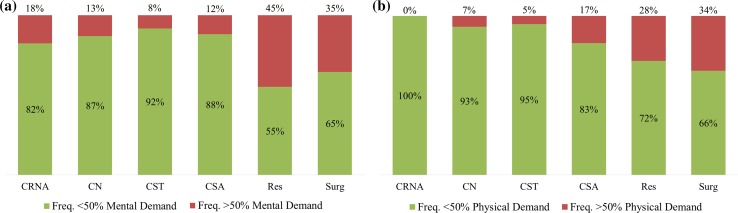
Frequency participants reported mental (**a**) and physical (**b**) demands over 50 % (high risk) by roles

**Fig. 4:**
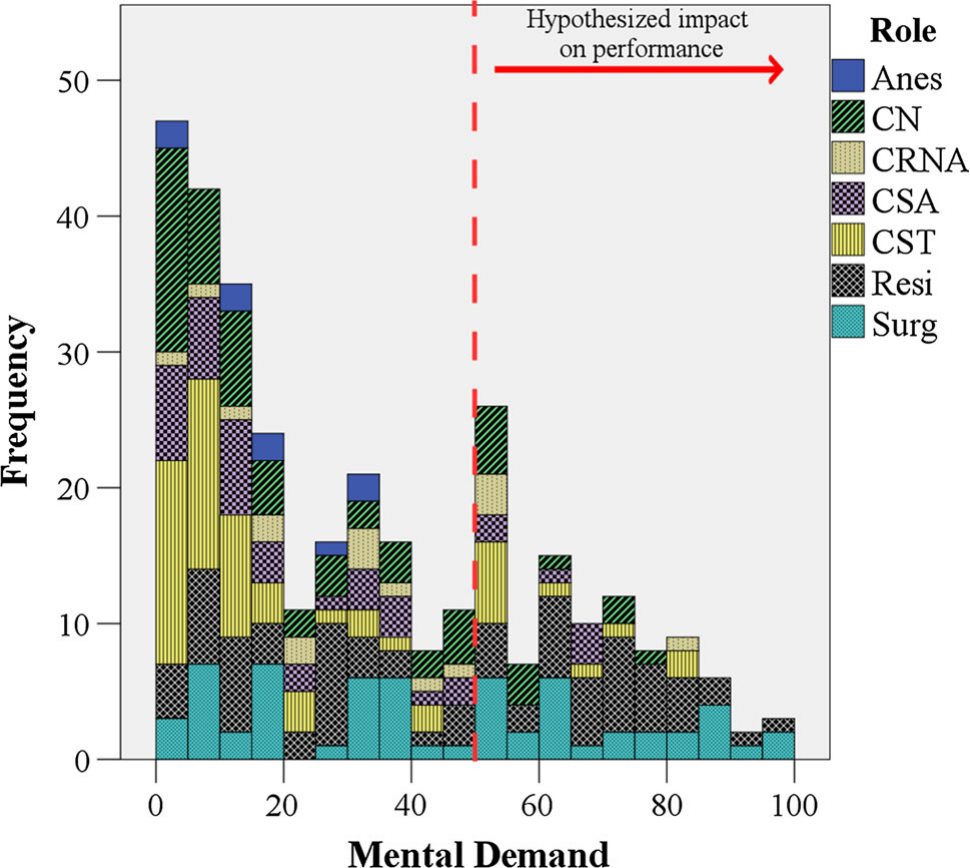
Distribution of mental demand across surgical team member roles with 50 score threshold adapted from workload studies indicating hypothesized impact on performance [35]

Although the exact thresholds of workload that lead to decrements in surgeon health and patient safety are still much debated [42, 44, 45], investigators have tentatively suggested that workload scores over 50–55 lead to increased performance errors and physical demand scores over 50 increased musculoskeletal injury risks [34–36, 38]. Adapting these suggested workload limits to our mental demand subscale data (Fig. 3a), residents exceeded the threshold most frequently (45 % of the time), followed by surgeons (35 %), CRNAs (18 %), CNs (13 %), CSAs (12 %), and CSTs (8 %). Distribution of mental demand ratings across teams is more left-skewed for CSTs and CNs than surgeons and residents (Fig. 4). For physical demand scale (Fig. 3b), surgeons exceeded the 50 % threshold in 34 % of their cases, followed by residents (28 %), CSAs (17 %), CNs (7 %), and CSTs (5 %).

## Discussion

This study quantified workload across surgical team member roles in the OR and demonstrated that the questionnaire is a responsive tool for detecting differences in intraoperative workload for variations in case complexity (basic versus advanced cases) and between different surgical team member roles. The following sections will discuss the implications of observed workloads (both high and low) among surgical team members.

### Mental demand

Comparing intraoperative workload across the surgical team, the surgeon reported the highest workload for each questionnaire subscale except mental demand, which was the highest for surgeons in training (residents). The mental and physical demands reported by surgeons were consistent with previously published ranges [11, 22, 39, 41, 46, 47].

High cognitive and physical workloads can impact surgeon performance and patient safety. Studies using NASA-TLX or SURG-TLX workload scores suggest a positive relationship between mental workload and performance errors [14, 37]. Using the individual subscales in the NASA-TLX, Yurko et al. found that increased mental and physical demands was associated with increased tissue damage on a porcine model and decreased suturing performance among novices [14]. Even though the exact thresholds of workload that lead to decrements in surgeon health and patient safety are much debated [42, 44, 45], investigators tentatively observed that mental demand was a major source of workload and NASA-TLX workload scores over 50 lead to increased errors and scores over 55 were predictive of performance declines during clinical tasks [35, 36]. Adapting this threshold to our data, surgeons and residents mental demand scored above the threshold for 35–45 % of cases (Fig. 3a). High mental demands may impact patient safety, and cognitive factors, e.g., confirmation bias and channeled attention on a single issue, are leading contributors to surgical never events [48]. Additional work is needed to validate the workload thresholds in a surgical setting. However, the suggested relationship reflects mechanisms explained by the Yerkes–Dodson or Frank–Starling Laws [49], and therefore, we believe are a reasonable starting point for analysis and interpretation of the data.

Mental demands for surgical team members outside the operative field, e.g., CRNAs, CSTs, and CNs, were observed to be in the red zone 18, 13, and 8 % of surgeries, respectively (Figs. 3, 4). Studies have suggested that lower mental workload reduced awareness and engagement, associated with increased frequency of performance impairing distractions (e.g., case-irrelevant conversations), and hypothesized to impact patient safety [49–51]. However, it is important to note that low mental workload implies only that the workload is lower compared to other high workload situations encountered by that individual and is therefore not necessarily a negative finding. In theory, achieving an optimal mental workload across the surgical team can prevent over- and under-loading the mental capacity of any single surgical team member. Our findings suggest that CSAs, CSTs, and CNs may have additional capacity to assume increased cognitive responsibilities in the OR. With an engaged and consistent surgical team, this available additional capacity could potentially be used to buffer the fatigue of other team members, reduce sentinel events, and improve patient safety [6, 7].

### Physical demand

A previous investigation in cardiac surgery and clinics suggested that surgeon’s intraoperative stress was primarily due to mental exertion [52]; however, we found that physical demand was as high as mental demand in our 78 cases. For team members within the operative field (i.e., surgeons, residents, and CSAs), physical workloads were not different and findings are consistent with previous work [47]. For team members outside the operative field (e.g., CSTs, CNs, and CRNAs), results showed that: (1) CSTs reported lower demands than CSAs, residents, and surgeons and (2) surgeons and residents reported higher demands than CRNAs, CSTs, and CNs. Physical demand differences may be due to task and equipment constraints among the roles. Specifically, the CRNAs and CNs occasionally performed their tasks in a seated position, and their postures and changes in their postures were not as constrained by surgical equipment and workplace layout while the postures of CSAs, residents, and surgeons were often more restricted. Tasks and workplace constraints may increase physical fatigue and lead to measured differences in physical demands [24].

An unexpected finding was the significantly lower physical demands experienced by CSTs since previous studies observed that scrub nurses/CSTs are exposed to significant OR ergonomic concerns, e.g., torso rotation, prolonged standing [15, 17, 18]. The low physical demand for CSTs observed in our study suggests that the CSA role in this institution offsets some of the work demands of CSTs of other institution, e.g., retracting, instrument holding. This transfer of physical workload from CSTs to CSAs may explain the higher physical demands reported by CSAs (Fig. 2b) and may increase CSA’s exposures to musculoskeletal injury risks. Specifically, previous studies observed that exposures to physical ergonomics risk factors in the OR doubled the odds ratio for musculoskeletal pain and symptoms [31], and median physical demand scores of 60 % (IQR 50–75 %) were associated with residents performing laparoscopic peg transfer tasks at “imminent risk for injuries” as assessed with the validated Rapid Upper Limb Assessment tool [38, 53]. Although the dose–response relationship between work exposures and musculoskeletal injuries in surgery is unknown, the intraoperative workload provides quantitative means to assess the level and frequency surgeon workload exposures and can be used to build an injury risk model that can link workload exposures to the high frequency of musculoskeletal symptoms reported in surgery [19, 24, 31, 54].

### Effect of technique on workload

Early studies found that mental strain and physical workloads during MIS techniques were higher than open techniques [24, 27, 55, 56]. Although estimated marginal means were slightly higher for MIS than open, physical demand (*p* > 0.20), mental demand (*p* > 0.80), complexity (*p* > 0.80), and distractions (*p* > 0.90) did not approach statistical significance in our sample size of 55 open and 21 MIS cases. The observed lack of significance is consistent with the workload ratings published recently by Weigl et al. [37]. It is important to note that only 37 participants in our study performed both MIS and open procedures in their practice, and further paired analysis on participants who performed both MIS and open procedures showed that mental demand, physical workload, distractions, complexity, and difficulty did not significantly differ between the two techniques. In contrast to the early studies comparing workload between open and MIS [24, 27, 55, 56], the results from the present study may indicate that the self-reported differences in workload between open and MIS experienced by the surgeons and surgical team have been reduced. However, this trend may be due to open techniques being chosen for more difficult cases, and further multi-institutional studies are warranted to investigate.

## Limitations

Although results only reflect the roles and specialties practiced in one medical institution, we believe that the findings are generalizable to other institutions. For example, workload measured for CSAs may reflect demands experienced by medical students or physician assistants at other institutions. Although surgical specialty did not significantly impact workload, this study was not designed to definitely test the impact of specialty and additional research is warranted to investigate whether workload imbalance is a global phenomenon or if it varies by specialty. While this study identifies role-specific trends in workload, further research is needed to identify specific causes for high physical and mental demands, case complexity, distractions, and degree of surgical difficulty in order to develop interventions to address these concerns. Although our response rate was satisfactory, questionnaires are subjective, voluntary, and from a convenience sample. In addition, while surgeons and residents typically stay for the entire procedure without breaks, other roles may rotate in-and-out of the OR, and the current questionnaire and methodology need further refinement to capture data for these transitions. Frequent OR rotation and supervision of multiple cases also contributed to anesthesiologist participation, thus, our study was not powered to detect differences between anesthesiologists and other roles.

## Conclusion

This study quantified workload variations based on procedure and team role during surgery. The questionnaire was responsive to differences in intraoperative workload across the surgical team over multiple surgical techniques and specialties. Since the survey uses validated SURG-TLX and GOALS subscales, we are able to compare results to other studies. It provides quantitative metrics for clinicians, engineers, and administrators to identify workload limitations and performance concerns in the OR. Findings suggest that modification of team member responsibilities, OR equipment, and/or workplace design may be needed to reduce the high mental and physical demands reported by CSAs, surgeons, and residents. Additionally, CSAs, CSTs, and CNs may have additional cognitive capacity that could be used to more optimally distribute workload. Lastly, this questionnaire tool can be used to monitor potential increases or imbalance in workload as the result of innovations in robotic or laparoscopic technologies.
